# Liver biopsy for assessment of chronic liver diseases: a synopsis

**DOI:** 10.1007/s10238-022-00799-z

**Published:** 2022-02-22

**Authors:** Aqib B. Chowdhury, Kosha J. Mehta

**Affiliations:** 1 GKT School of Medical Education, Faculty of Life Sciences and Medicine, King’s College London, London, UK; 2 Centre for Education, Faculty of Life Sciences and Medicine, King’s College London, London, UK

**Keywords:** Liver biopsy, Ishak, METAVIR, Batts-Ludwig, Alcoholic hepatitis histology score (AHHS), NAFLD activity score (NAS), MAFLD, Repeat biopsy

## Abstract

The world-wide increase in chronic liver disease (CLD) calls for refinement of diagnostic and prognostic measures for early and accurate disease detection and management. Regardless of the aetiology, liver biopsy allows direct visualisation of specimen under the microscope. It facilitates histological evaluation of disease-specific morphological alterations. Thereby, it aids in disease diagnosis, prognosis, and assessment of treatment compliance/response. Indeed, with the advent of non-invasive methods, liver biopsy is used less frequently than before, but it is still considered as a gold standard for staging and grading several CLDs. This short review revisits liver biopsy. It highlights the significance of liver biopsy in evaluating CLDs and explains the commonly used Ishak, METAVIR and Batts-Ludwig scoring systems for grading and staging CLDs. The utility of liver biopsy in examining alcohol-related liver disease and non-alcoholic fatty liver disease (NAFLD) is discussed along with the disease-specific alcoholic hepatitis histology score (AHHS) and non-alcoholic fatty liver disease activity score (NAS). Additionally, the review elaborates on the role of liver biopsy in evaluating viral hepatitis, haemochromatosis, and hepatocellular carcinoma. Contextual explanation on the diagnosis of metabolic dysfunction-associated liver disease (MAFLD) is provided. The significance and clinical indications of repeat biopsy are also explained. Lastly, caveats and limitations associated with liver biopsy are reviewed. Essentially, this review collates the application of liver biopsy in assessing various CLDs and provides succinct explanations of the core scoring systems, all under one roof. It is clinically relevant and provides a useful synopsis to budding scientists and hepato-pathologists.

## Introduction

Chronic liver disease (CLD) includes pathologies arising from various aetiologies such as viral hepatitis (B and C), alcohol-related liver disease (ARLD), non-alcoholic fatty liver disease (NAFLD)/non-alcoholic steatohepatitis (NASH), haemochromatosis, autoimmune disease, Wilson’s disease and alpha-1 anti-trypsin deficiency [1]. Independent of the aetiology, CLD pathology generally involves a sequela of several overlapping stages that show no clear demarcation. Generally, these stages showcase an increasing pathological gradient and include hepatic steatosis (fatty liver) followed by steatohepatitis (fatty liver and concomitant inflammation) or hepatitis (inflammation, that can occur independent of steatosis), fibrosis (excessive deposition of extracellular matrix) and cirrhosis (scarring). The latter is a late-stage liver condition that can progress to life-threatening complications such as portal hypertension, liver failure or hepatocellular carcinoma (HCC) [2].

CLD accounts for more than 2 million deaths annually [3] with 1.3 million deaths reported in 2017 [4]. However, early disease diagnosis together with appropriate disease management that monitors disease progression in response to treatments can halt, decelerate, and at times, reverse the pathological progression of CLD, thereby reducing the possibility of decompensated cirrhosis, HCC, and/or liver failure. Therefore, appropriate diagnostic and prognostic measures are important as these can ultimately enhance patient outcomes and curb CLD-associated morbidity and mortality [5, 6].

Despite the utility of serum markers and non-invasive imaging techniques like elastography, liver biopsy, followed by histological examination, remains the gold standard for measuring and staging liver fibrosis and for diagnosing cirrhosis [7–9].

Accordingly, this review addresses the biopsy-based Ishak, METAVIR and Batts-Ludwig scoring systems that are used for grading and staging CLDs. In addition, it discusses the disease-specific alcoholic hepatitis histology score (AHHS) and NAFLD activity score (NAS). Further on, it highlights the significance of liver biopsy in assessing viral hepatitis, haemochromatosis, and hepatocellular carcinoma. Finally, the caveats and limitations of using liver biopsies for disease assessment are addressed.

## Significance of liver biopsy in CLD assessment

Liver biopsy facilitates direct visualisation of the absence or presence of hepato-pathology. It is probably the only method currently available that definitively distinguishes between the different pathological stages of CLD and helps identify factors associated with disease progression. For example, liver biopsy is essential for fibrosis measurement in NAFLD and NASH [7]. Also, it distinguishes between bland fibrosis and fibrosis associated with necroinflammation [7]. Thereby, it helps not only in disease diagnosis but also in assessing disease severity [10, 11]. As such, fibrosis is primarily a histological measurement and liver biopsy is the only way of histological assessment. Importantly, for disease management, detailed knowledge of the fibrosis stage is required because it precedes the more advanced cirrhosis stage and therefore offers a better chance and degree for disease regression following intervention. No wonder, with every increase in the stage of fibrosis, there is a lesser chance of transplant-free survival, at least in case of NAFLD [7].

Moreover, while in non-invasive methods, interpretations could vary depending on co-existing diseases, interpretations of liver biopsy specimen via the commonly used scoring systems are universally accepted and can provide reliable information regardless of the presence of co-existing disease [7]; for example in diagnostic dilemmas involving co-existing disorders like steatosis and HCV infections or in distinguishing between autoimmune hepatitis and NASH in obese individuals presenting abnormal liver function tests and positive autoimmune serology [11, 12]. Indeed, liver biopsy specimen can aid in the pathological evaluation of autoimmune hepatitis [13] and in determining disease severity and stage in chronic hepatitis B and C infections; although not essential for diagnosis [14]. Interestingly, while the non-invasive approaches are more popular, these modalities have been validated by using the results of liver biopsy as a reference standard [7]. Essentially, liver biopsy can be used to ascertain diagnosis, stage and grade the disease, for prognostication of an already diagnosed liver disease, and to develop a disease management plan [8, 11]. An overview of the methods of liver biopsy is provided in Table 1.

**Table 1:** Methods of performing liver biopsy- an overview

Method	Characteristics
Percutaneous biopsy	It is commonest type of biopsy whereby a needle is directly inserted into the liver through the skin to obtain a small sample. The process is usually guided by an ultrasound or CT scan [16]
Transvenous biopsy	It is also used frequently, particularly in individuals that cannot undergo percutaneous liver biopsy; for example, in those with significant ascites or those who are morbidly obese [12] or those presenting thrombocytopenia or coagulopathy [8]. The process involves inserting a catheter in either the jugular (transjugular biopsy) or femoral vein (transfemoral biopsy) which is then passed into the hepatic vein. Thereafter, a biopsy needle is threaded through the catheter and a sample is taken from the liver
Laparoscopy liver biopsy	It is performed when transvenous biopsy has failed or in patients with severe coagulopathies where biopsy of a focal lesion is required. It is done using conventional laparoscopy through the abdomen [16]
Plugged biopsy	It is a modified form of percutaneous biopsy. It can be used in patients with impaired coagulation, ascites or when transvenous biopsy has failed. This technique involves using a coaxial introducer needle within the liver from which an inner trocar is removed. Thereafter, samples are taken using a cutting needle and the biopsy track is plugged, usually using a gelatine sponge [16]. Independent of the approach, a sample of at least 1.6 cm length and 1.2–1.8 mm in diameter containing approximately 10 portal tracts is considered adequate for analysis [17]

There are four ways of performing a liver biopsy: percutaneous, transvenous, laparoscopic and plugged biopsy [12]. Samples are analysed by pathologists who diagnose, stage, and grade the liver disease based on histological features/findings; details of which are elegantly reviewed elsewhere [8, 11, 15]

## Staging and grading CLDs: Ishak, METAVIR and Batts-Ludwig scoring systems for evaluating disease severity via liver biopsy

Various scoring systems have been developed to stage and grade the histological samples obtained from liver biopsy. These can be used regardless of disease aetiology. Here, stage refers to the level of liver fibrosis and indicates how far the disease has progressed along its natural history. Grade refers to the level of necroinflammation and hepatocellular injury, which reflects the rate of disease progression as well as disease severity [8]. Thus, in evaluating disease severity, these scoring systems focus primarily on two aspects: inflammation (hepatitis) and fibrosis. Together, staging and grading can help in the diagnosis, prognosis, and therapeutic management of CLDs.

**Fig. 1:**
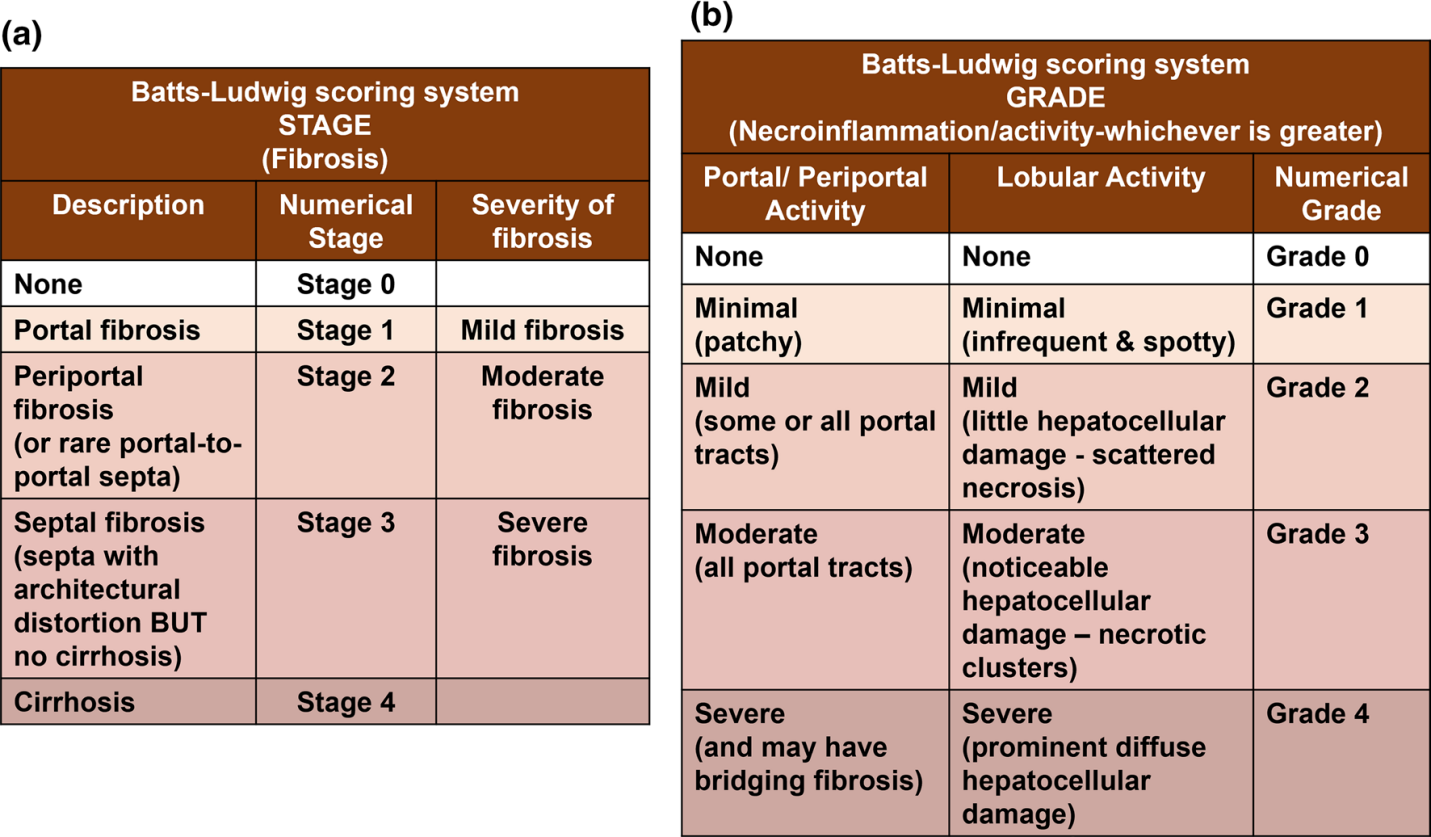
Batts-Ludwig scoring system. The Batts-Ludwig scoring system is used for staging of fibrosis (**a)** and grading (**b)** of histological specimens obtained from the liver of patients with chronic hepatitis. Here, values of both stage (**a)** and grade (**b)** range from 0 to 4. (**a)** Staging is based on the presence of portal/periportal fibrosis and septa formation with/without cirrhosis, which correspond to stage 4, while (**b)** grading is based on either the portal/periportal activity or lobular activity. Here, whichever activity demonstrates greater severity is affirmed [11, 18] i.e. the overall grade is the one which is present at the greatest degree. The Batts-Ludwig system is also known as the modified Scheuer system. Tables adapted from Rockey et al. [11] and Batts Ludwig [18]

**Fig. 2:**
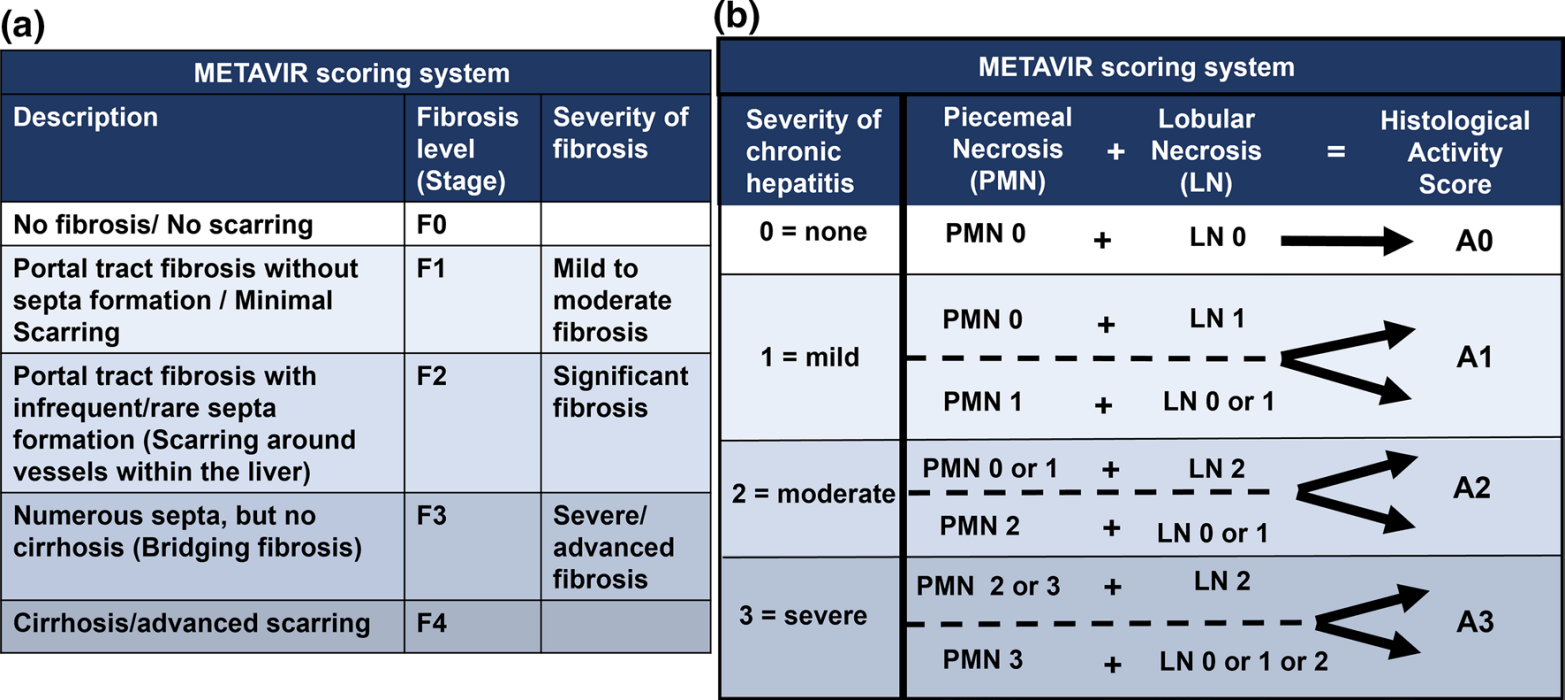
METAVIR scoring system. The METAVIR (Meta-analysis of Histological Data in Viral Hepatitis) scoring system is used for the assessment of histological samples obtained from a liver biopsy. It assesses fibrosis level (**a)** and histological activity score (**b)**, reflecting disease stage and grade, respectively. Here, fibrosis level (**a)** ranges from F0 (no fibrosis) to F4 (cirrhosis) and is primarily based on the presence of fibrosis in the portal tract and the number of septa (fibrous bands of tissue). Level F2 or higher is considered as significant fibrosis, while F3 or higher is considered as advanced fibrosis [9]. The histological activity score (b) is generated by the METAVIR algorithm which constitutes no activity (A0), mild activity (A1), moderate activity (A2) and severe activity (A3). This activity score is generated by combining the degree of piecemeal necrosis (PMN) (interface hepatitis) and lobular necrosis (LN) in the liver specimen [19, 20]. PMN is the necrosis of the periportal zone (as often seen in autoimmune and viral hepatitis) characterised by inflammation that extents from portal tract to periportal zone and damaged periportal hepatocytes [21]. It essentially reflects inflammation and destruction of hepatocytes bordering the portal tracts. PMN 0,1,2 and 3 correspond to none, mild, moderate, and severe, respectively. On the other hand, LN involves necrosis of liver lobule and can range in severity from focal necrosis (group of necrotic hepatocytes with infiltrated inflammatory cells in an area) to bridging necrosis (confluent necrosis that links terminal venules to portal tracts) [22]. LN 0,1 and 2 correspond to none or mild, moderate, and severe, respectively. Tables adapted from Rockey et al. [11], Theise [20], and Bedossa and Poynard [19]

**Fig. 3:**
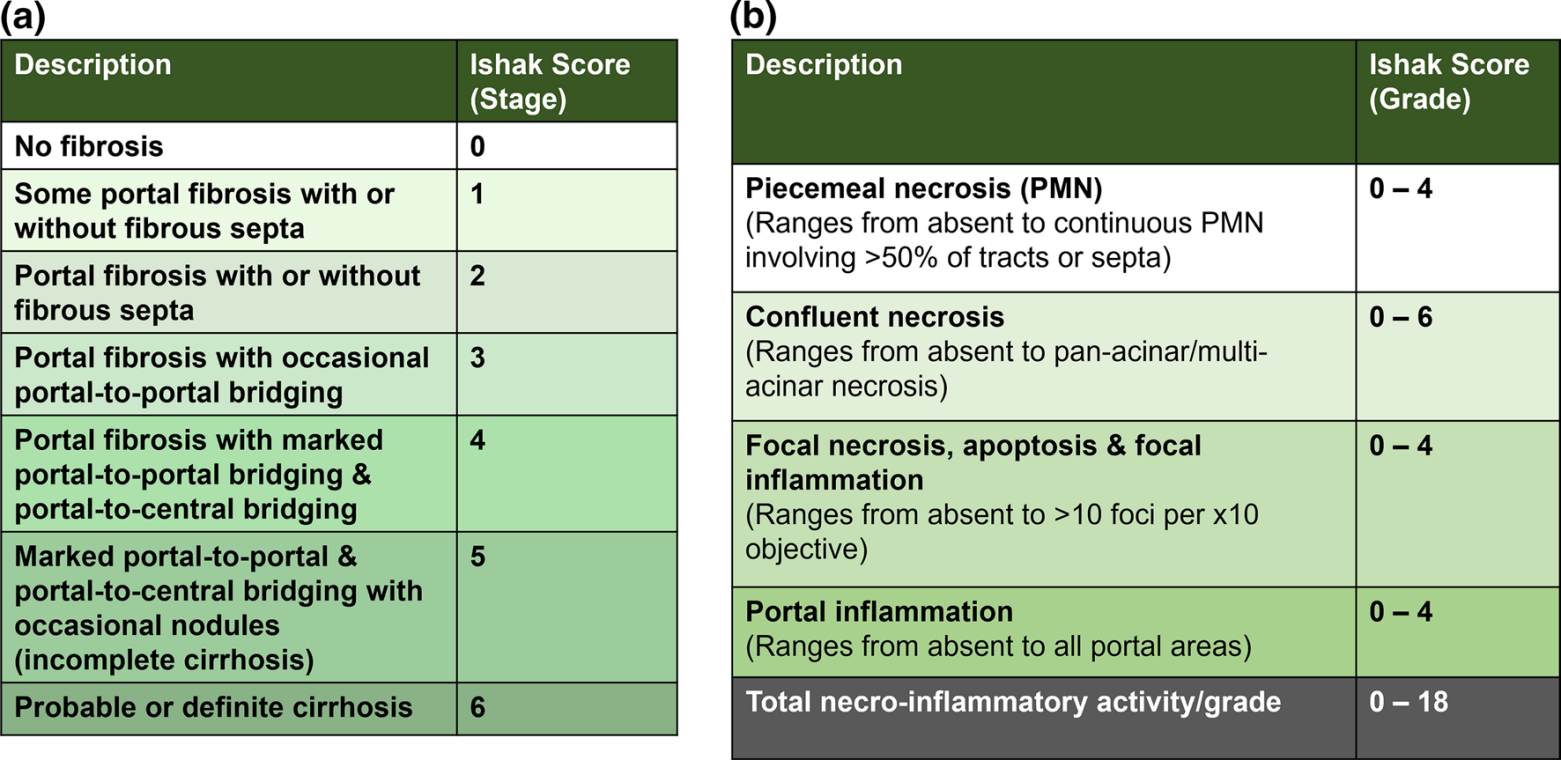
Ishak scoring system. The Ishak system [23] is also known as the modified Knodell or modified histology activity index (HAI) score. It assesses the level of fibrosis (**a)** where scores range from 0 (no fibrosis) to 6 (cirrhosis). Here, levels F5 and F6 reflect incomplete and definite cirrhosis, respectively [9]. This system also helps ascertain the levels of necro-inflammatory activity through grading (**b)**. It assesses the level of piecemeal necrosis (score 0–4), confluent necrosis (score 0–6), focal necrosis, apoptosis, and focal inflammation (score 0–4), and portal inflammation (score 0–4). Severity of each parameter is assessed, and the overall score for grading could be given between 0 and 18 [23]. Further details reviewed by Theise [20]. Tables adapted from Ishak et al. [23] and Theise [20]

**Fig. 4:**
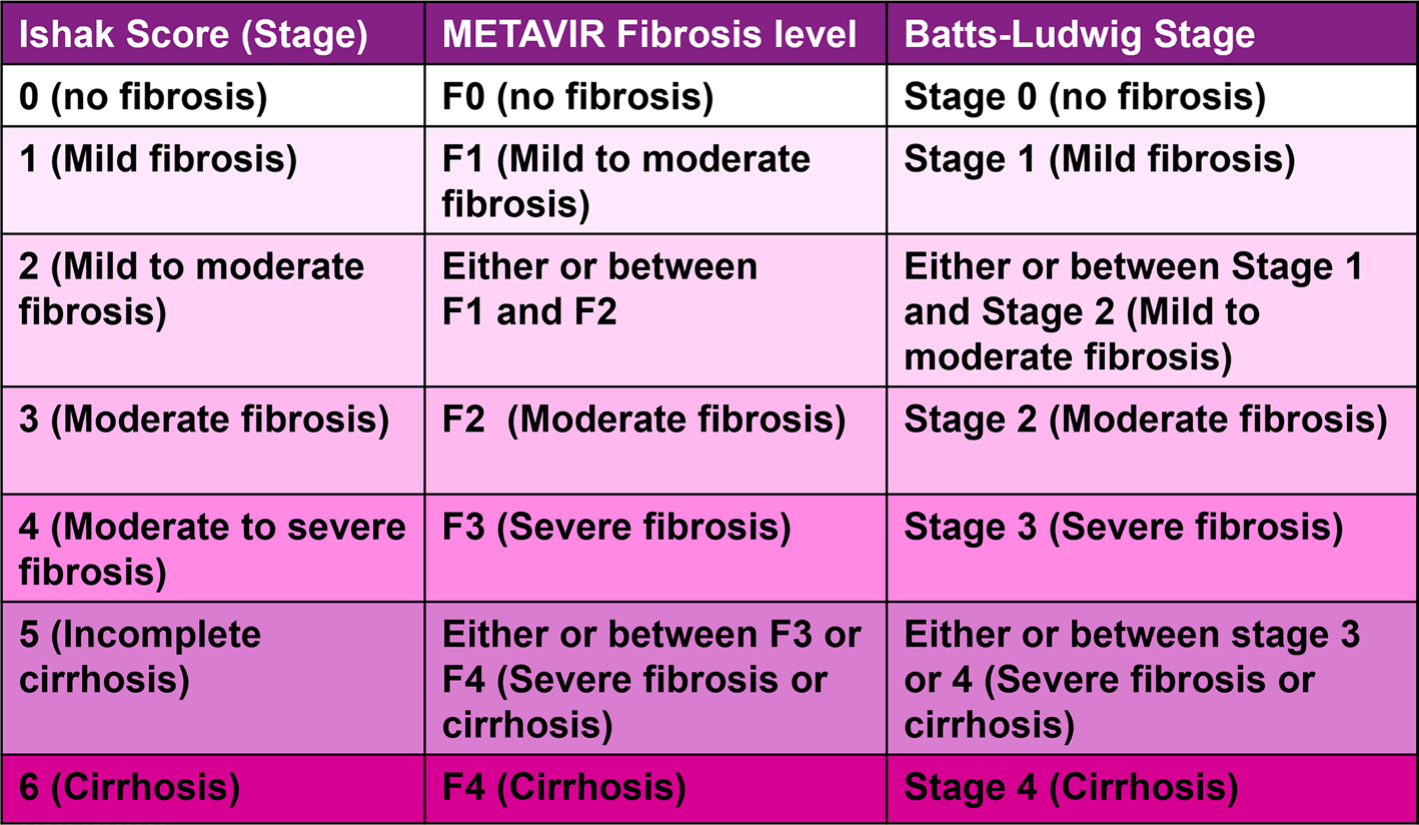
Proposed correspondence and comparison between Ishak, METAVIR and Batts-Ludwig scoring systems. The figure proposes a possible correspondence in pathological stages between the three systems. The Ishak staging system provides more descriptive and comprehensive information on fibrosis than the METAVIR and Batt-Ludwig systems. A single METAVIR fibrosis level or Batts-Ludwig stage constitutes multiple Ishak scores and there are several Ishak scores between the METAVIR fibrosis levels and Batts-Ludwig stages. In the METAVIR and Batt-Ludwig systems, cirrhosis is stage 4, unlike the Ishak system where cirrhosis is represented at stages 5 and 6. Although the Ishak score provides more information on fibrosis, due to its complexity, it is less reproducible and not easily applicable in clinical practice

The Batts-Ludwig, METAVIR and Ishak scoring systems have been detailed in Figs. 1, 2 and 3, respectively. Figure 4 compares these systems.

Due to its complexity the Ishak system is less reproducible than METAVIR and Batts-Ludwig scoring systems and is not easily applicable in regular clinical practice. Thus, the American Association for the Study of Liver Disease (AASLD) recommends that such complex scoring systems would be appropriate for clinical trials that involve large cohorts of patients, while the comparatively simpler systems like Batts-Ludwig and METAVIR would be more appropriate for day-to-day clinical practice when dealing with individual patients [10, 11].

A study compared these systems by using the kappa-(*k*)-coefficient. *k*-coefficient is a statistical measure of agreement (or equal variability) used for qualitative/categorical items and is typically used to assess inter-observer and intra-observer agreement. It can also be used between two qualitative scoring systems and the values range from 0 to 1. These values are typically interpreted as follows- 0: no agreement, 0–0.2: slight agreement, 0.21–0.4: fair agreement, 0.41–0.6: moderate agreement, 0.61–0.8: substantial agreement and 0.81–1: almost perfect agreement [24].

The assessment of concordance between the METAVIR and Ishak systems revealed a near perfect correlation (*k* = 0.998) with regards to fibrosis and substantial correlation (*k* = 0.627) between the necroinflammatory scores when evaluating the histological specimens from 82 patients with serologically confirmed chronic viral hepatitis [25]. The reason for the comparatively lower concordance in necroinflammatory scores could be because Ishak score takes piecemeal necrosis, confluent necrosis, focal necrosis, apoptosis, and portal inflammation into consideration, which is considerably more comprehensive than the METAVIR activity score that takes into account only piecemeal and lobular necrosis (Figs. 2 and 3).

## Liver biopsy for liver pathologies

### Alcohol-related liver disease (ARLD)

ARLD shows an alcohol-induced pathological spectrum ranging from hepatic steatosis to alcoholic hepatitis and then alcohol-associated cirrhosis that could lead to liver failure or hepatocellular carcinoma. While ARLD is an independent cause of liver disease, it also facilitates the progression of NAFLD, viral hepatitis, haemochromatosis, and other liver conditions. The approach to tackle ARLD is “screening, brief intervention and referral to treatment”. Alcohol biomarkers such as liver enzymes, bilirubin, gamma-glutamyl transferase, carbohydrate-deficient transferrin, ethyl glucuronide, and phosphatidylethanol can aid in ARLD diagnosis [26].

Biopsy is not commonly used for ARLD assessment (alcoholic steatosis). It is utilised in rare ARLD cases when clinical presentation and serological tests are unclear or when there are co-existing liver conditions making the clinical presentation more complex and ambiguous [27, 28]. Histological features of alcoholic hepatitis (and also cirrhosis) include neutrophilic lobular inflammation, degeneration of hepatocytes (Mallory-Denk bodies and ballooning), steatosis and fibrosis [26].

**Fig. 5:**
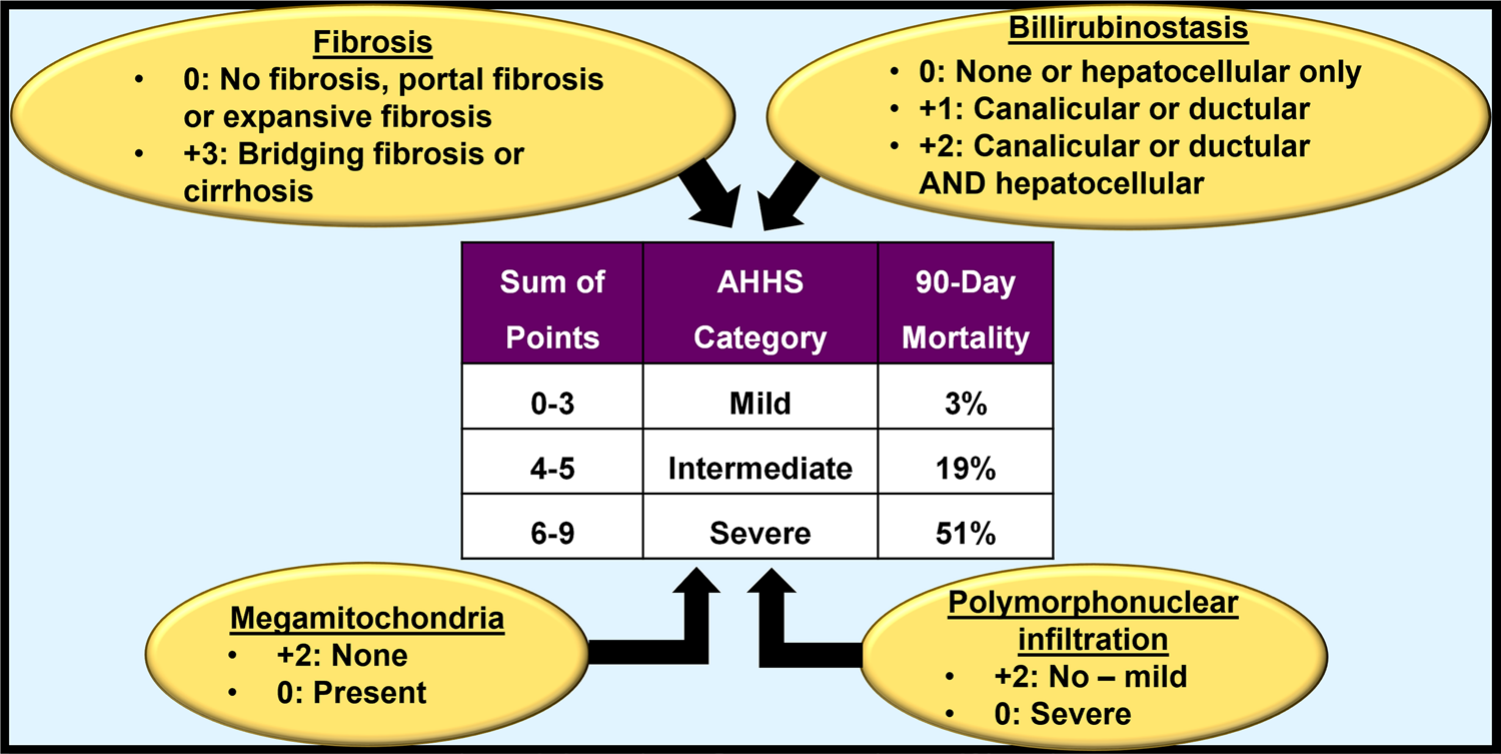
Alcoholic Hepatitis Histological Score (AHHS). The AHHS individually assessed and scored the level/severity of four histological features associated with ARLD, namely, fibrosis, bilirubinostasis, megamitochondria and polymorphonuclear infiltration. Scores were given for each histological feature, which made the total score range from 0 to 9, and the severity category was determined accordingly. Based on the total score achieved, patients were designated into three categories: mild (scores 0–3), intermediate (scores 4–5) and severe (scores 6–9). This categorisation allowed physicians to predict different risks of death within 90 days for the three different groups [29]

In the absence of well-validated non-invasive methods to diagnose alcoholic hepatitis, the biopsy-based alcoholic hepatitis histology score (AHHS) was proposed and validated for prognostic stratification of alcoholic hepatitis. It assessed the severity of four histological features: bridging fibrosis or cirrhosis (i.e. degree of fibrosis), type of billirubinostasis, polymorphonuclear infiltration (i.e. degree of neutrophil infiltration) and presence of megamitochondria (Fig. 5). Notably, AHHS by itself does not allow the staging and grading of ARLD. It is useful for predicting mortality [29]. However, recently, Dubois et al. observed that AHHS did not corelate with 1,3 and 6-month survival and could not predict short-term survival in patients with severe alcoholic hepatitis [30].

### Non-alcoholic fatty liver (NAFL)/Non-alcoholic steatohepatitis (NASH)

Non-alcoholic fatty liver disease (NAFLD) is characterised by hepatic steatosis i.e. > 5% fat accumulation in the liver (determined either by histology or imaging) that occurs in the absence of secondary causes of liver fat accumulation such as alcohol consumption, usage of steatogenic medication or monogenic hereditary disorders.

Distinct from ARLD, in NAFLD patients, liver biopsy is used to diagnose and assess fibrosis and it remains the gold standard for NASH diagnosis [17, 31] and for evaluation of fibrosis in NAFLD patients [7]**.** However, AASLD recommends that only those NAFLD patients should be offered a liver biopsy who present a high risk of steatohepatitis and/or advanced fibrosis (for example those with co-existing metabolic syndrome, elevated aminotransferases, individuals over 60-years-old) or when co-existing liver disease cannot be ruled out without a liver biopsy [32]. In patients with suspected NAFLD, liver biopsy should be considered when serum ferritin and iron saturation are high. This would assess the presence and degree of hepatic iron accumulation [31].

Histologically, NAFLD can be divided into non-alcoholic fatty liver (NAFL) (~ 70–75% of NAFLD patients) and it’s progressive subtype non-alcoholic steatohepatitis (NASH) (~ 25–30% of NAFLD patients) [5]. NAFL (also known as simple steatosis) is ≥ 5% steatosis with or without mild non-specific inflammation and without hepatocellular injury (hepatocyte ballooning). In contrast, NASH is ≥ 5% steatosis with lobular inflammation and hepatocyte ballooning with or without fibrosis. In NAFL, progression to cirrhosis is rare (< 4%), whilst up to 20% of those with NASH progress to cirrhosis. Currently, biopsy is the only way to reliably and definitively diagnose and stage NAFLD, and distinguish between NAFL and NASH [5, 31, 32].

**Fig. 6:**
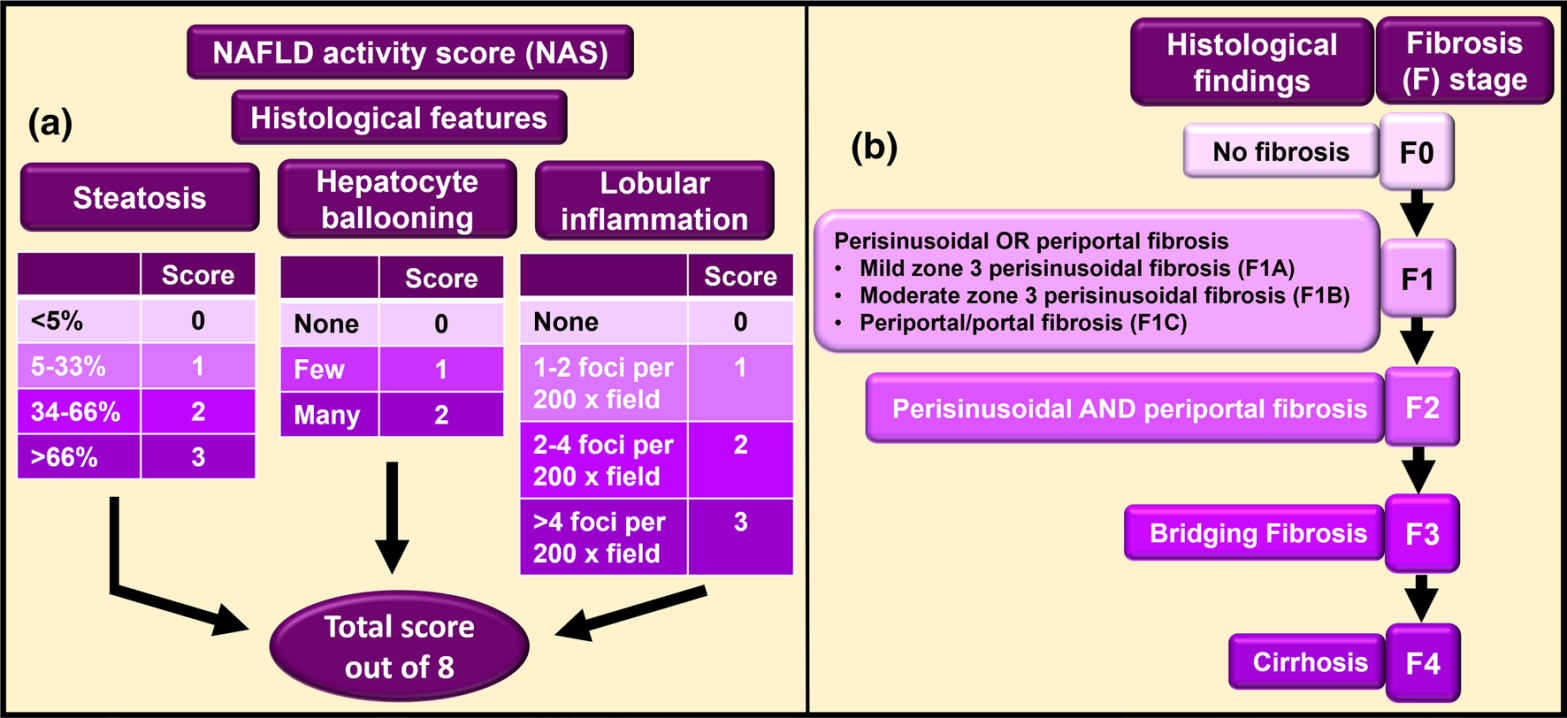
NAFLD activity score (NAS) and staging of fibrosis in NAFLD. A standardised semiquantitative histological scoring system for NAFLD is used in clinical practice. NAFLD Activity score (NAS) (**a)** is the sum of steatosis, hepatocyte ballooning and lobular inflammation (other miscellaneous features are included). These are features of active injury. The total score (could be from 0 to 8) is deduced by adding the scores achieved in these three features. In addition to including both NAFL and NASH, NAS offers other advantages such as relative ease in usage and understanding and allowing hepato-pathologists to be able to provide a clear statement that the score is based on the three lesions/features. Also, this system is particularly useful in monitoring disease progression because the features of active injury described under NAS have been found to be reversible with treatment. NAS does not assess fibrosis. Therefore, usage of a separate fibrosis staging system (**b)** in conjunction with NAS has been proposed to obtain a wider picture of pathology [34]. Information adapted from Kleiner et al. [34]

Brunt et al. proposed a staging and grading system specifically for NASH [33]. Since this system did not overarch NAFLD, which includes both NAFL and NASH, the Pathology Committee of NASH Clinical Research Network proposed, developed, and validated a semiquantitative scoring system for histological specimens from those with suspected NAFLD. This is currently used in clinical practice and is referred to as the NAFLD Activity Score (NAS), which includes both NAFL and NASH) [34] (Fig. 6). NAS ≥ 5 has been shown to be correlated with NASH diagnosis [34]. Therefore, some pathologists used this to diagnose NASH rather than basing diagnosis on histological findings [35]. But NAS was originally developed to be used as an adjunct to the histological diagnosis and to only grade and stage NAFLD, not diagnose NASH; essentially, to examine treatment-induced alterations rather than diagnosis [17]. This unintended use of NAS was evaluated by Brunt et al. Data revealed that 7% of those considered to have no NASH by the pathologists’ histological assessment had NAS ≥ 5 [35]. Thus, the usage of NAS ≥ 5 as a surrogate for the diagnosis of NASH can lead to false positives.

There are similarities between the histological features of NASH and ARLD, namely, steatosis, hepatitis and steatohepatitis as well as the features of hepatocyte injury including hepatocyte ballooning, apoptosis, necrosis and fibrosis. Thus, biopsy cannot distinguish between alcohol-associated steatohepatitis and NASH [26]. In clinical practice a corroborating history of alcohol abuse and appropriate clinical features can help differentiate between the two.

However, there are some differentiating features between the two (NASH and ARLD). Firstly, in ARLD, lobular inflammation is commonly presented as lesions known as satellitosis, which are clusters of polymorphonuclear cells. Additionally, steatosis is not always present in ARLD, but thickening and fibrosis of terminal hepatic venules and veno-occlusive lesions have been described. Sclerosing hyaline necrosis has been found to be exclusive to alcoholic hepatitis [17].

### MAFLD: metabolic dysfunction-associated fatty liver disease

Metabolic disease is on the rise [36]. Further understanding of NAFLD pathogenesis has indicated that it originates from an underlying state of metabolic dysfunction [37, 38]. Therefore, usage of a new overarching term metabolic dysfunction-associated fatty liver disease (MAFLD) has been proposed to encompass the full spectrum of the disease [39].

MAFLD is defined as the presence of hepatic steatosis (confirmed by presence of serum biomarkers of fatty liver, histological analysis, or imaging) along with one of the following three metabolic features: overweight or obesity (BMI > 25 kg/m^2^ in white and > 23 kg/m^2^ in Asian persons), confirmed type-2 diabetes mellitus or demonstration of lean or normal weight but with the evidence of metabolic dysregulation. Here, metabolic dysregulation implies the presence of at least two metabolic risk factors from the following: waist circumference (≥ 102/88 cm in white men and women or ≥ 90/80 cm in Asian men and women), prediabetes (defined as fasting plasma glucose of 5.6–6.9 mmol/L), inflammation with increased levels of high-sensitivity serum C-reactive protein (hs-CRP) (> 2 mg/L), increased blood pressure (≥ 130/85 mmHg) or on drug treatment, reduced HDL-cholesterol levels (< 1.0 mmol/L for men and < 1.3 mmol/L for women) or on drug treatment, elevated plasma triglyceride levels (≥ 1.70 mmol/L) or on drug treatment, and homeostasis model assessment of insulin resistance (HOMA-IR) score ≥ 2.5. The development of MAFLD is influenced by factors like age, sex, ethnicity, dietary habits, endogenous metabolic status, gut microbiota, and genetic predisposition [39–41].

The diagnostic criteria for MALFD are more inclusive than that of NAFLD. The latter includes the presence of steatosis > 5% but neither acknowledges the presence of other CLDs nor the factor of excess alcohol intake. However, fatty liver disease may co-occur with other CLDs. In another perspective, MAFLD patients may show the presence of concomitant aetiology for liver disease [42]. Therefore, an investigation under MAFLD is clinically more appropriate and useful as it fosters more clinical suspicion, thereby lowering the probability of missed diagnosis.

Also, metabolic disorders including obesity and type-2 diabetes mellitus are characterised by low-grade systemic inflammation. So, the inclusion of hs-CRP as a marker of inflammation within the diagnostic criteria for MAFLD is extremely important and clinically relevant [41]. In routine clinical practice when checking for hepatic steatosis in lean non-diabetic patients with normal BMI, hs-CRP and HOMA-IR are not evaluated [42]. Inclusion of these criteria in MAFLD diagnosis can mediate and aid in the screening of this population.

Since this is a newly coined term, researchers are still studying its utility in real life situations. A study compared MAFLD and NAFLD characteristics amongst a population of patients with fatty liver disease. Data revealed that MAFLD patients were older, demonstrated higher levels of BMI, HOMA-IR, and lipid and liver enzymes, higher proportions of metabolic comorbidities like diabetes and hypertension, and higher frequency of advance fibrosis in those who consumed alcohol. It was concluded that the MAFLD definition allowed identification of those hepatic steatosis patients who were at high risk of disease progression [43]. Notably, while MAFLD diagnostic criterions exist, concerns are being raised over their practical applicability due to the lack of availability of data on some of the parameters in some instances [42].

MAFLD diagnosis is based on the presence of hepatic steatosis. Imaging techniques like ultrasound can be used to determine liver fat deposition in addition to using serum biomarkers of lipid accumulation. Liver biopsy can also be used for the assessment of hepatic steatosis, and it does allow more in-depth assessment of the condition compared to non-invasive alternatives. However, because of its invasive nature and associated complications, this approach should be reserved only for complex cases, for example when concurrent presence of another CLD is suspected. Regardless, just like other CLDs, MAFLD can be staged and graded to assess disease severity and progression [39]. However, it is noteworthy that the staging and grading systems rely heavily on the assessment of fibrosis, which is informed by inflammation. Liver biopsy produces only a single snapshot of the disease at one time-point from the long-lasting CLD. It does not take into account (unable to assess) alternations in inflammatory status i.e. chronic–relapsing or intermittent, as found in several CLDs [41] and this may well be the case with MAFLD too.

### Hepatitis B and C virus infections

Viral hepatitis is a result of liver infection with hepatitis A, B, C, D or E viruses. The clinical spectrum of hepatitis B and C infections generally include an inflamed liver (hepatitis) demonstrating acute and chronic infection phases, where the latter phase can progress to cirrhosis and hepatocellular carcinoma. While hepatitis C can be the deadliest virus, remarkable progress has been made in the treatment of hepatitis C infection. Hepatitis B remains a public health problem world-wide. Despite medical advances, it causes significant mortality, primarily due to subsequent cirrhosis and hepatocellular carcinoma [44, 45].

Liver biopsy is no longer essential or recommended for the diagnosis of chronic hepatitis B and C infections. However, it still remains the gold standard for prognostication, staging/grading, and therapeutic management, at least for a subset of patients where biochemical or serological markers show inconclusive results [15]. This difficulty is partly attributed to the challenges in determining disease activity and fibrosis via serum biomarkers, particularly in chronic hepatitis B infections [46]. Liver biopsy is also extremely useful in facilitating Orcein staining that can demonstrate the expression of hepatitis B surface antigen in the biopsy sample [8]. Moreover, liver biopsy is essential for studies of covalently closed circular DNA (cccDNA) in case of hepatitis B infection. Such studies are very useful because this viral cccDNA is the most important genomic form that determines the persistence of infection. In chronic hepatitis C infection, EASL recommends the usage of non-invasive methods to assess disease severity. Liver biopsy should only be reserved for those cases where either the non-invasive alternative has not shown reliable results or in cases of suspected or known mixed aetiologies or to determine fibrosis level in case of uncertainty or the potential presence of additional aetiologies [44, 45].

Histologically, chronic viral hepatitis patients show inflammation fibrosis/cirrhosis and hepatocellular alterations that characteristically include portal tract inflammation with mononuclear cells, interface hepatitis, focal lobular necrosis, and bile duct damage. Liver biopsy followed by histopathological assessment can reveal the level of necroinflammation and fibrosis, presence of lesions such as liver cell dysplasia, steatosis and hemosiderosis or presence of comorbid liver conditions [47]. In case of Hepatitis C infection, chronic hepatitis can be accompanied with steatosis and bile duct injury [8]. Determination of severity of necroinflammatory activity and fibrosis is important for prognosis and therapy. Fibrosis has proved to be a better predictor of disease progression than necroinflammation. These parameters are assessed using several scoring systems such as the Ishak, METAVIR and Batts-Ludwig classifications [47], although elastography is now well-established to assess fibrosis [8]. Usually, liver biopsy is indicated prior to starting the antiviral treatment [47]. However, in chronic hepatitis B infection, antiviral treatment can be started without liver biopsy (in patients with HBV DNA > 20,000 IU/mL and alanine aminotransferase level twice the upper limit of normal) because the benefits extend across all stages of fibrosis [45, 46].

### Hereditary haemochromatosis

Hereditary haemochromatosis, the iron-overload condition, is the most common genetic disorder amongst people of northern European origin. *HFE* gene mutations (C282Y and H63D) being the common cause, its pathophysiology involves increased absorption of dietary iron leading to excess iron deposition in various organs, and it is associated with the development of end-stage liver disease, diabetes, and cardiac disease [48].

Earlier, liver biopsy was considered as the only definitive approach for determination of tissue iron deposition. Following the advent of genetic testing of *HFE* gene mutations, early diagnosis (which evades the need for biopsy) and the usage of non-invasive approaches such as MRI that ascertain liver iron levels, liver biopsy is no longer necessary for diagnosing hereditary haemochromatosis. However, it is still useful for the following: to determine the presence of fibrosis/cirrhosis (thereby serving prognostic purposes [49, 50]), to stage fibrosis in C282Y homozygotes that have serum ferritin levels > 1000 ng/mL, to determine hepatic iron concentration for differentiating between C282Y homozygotes and compound homozygotes, and to exclude other CLDs like alcoholic and non-alcoholic fatty liver [50]. In C282Y homozygotes, if serum ferritin levels are less than 1000 ng/mL, then liver biopsy is not normally recommended unless the patient presents other risk factors for cirrhosis. If advanced fibrosis/cirrhosis is determined through other diagnostic approaches, then liver biopsy can be utilised [51]. Determination of cirrhosis is important because it predicts increased risk for hepatocellular carcinoma [52]. Moreover, liver biopsy plays an important role in the assessment of non-HFE haemochromatosis patients and when there is a discrepancy between HFE genotypes and iron assessments [53].

As expected, for hereditary haemochromatosis, liver biopsy is followed by histopathological evaluation that not only includes the regular Masson's trichrome and haematoxylin–eosin staining for fibrosis staging, but also Perls' Prussian blue staining to assess cellular distribution of hepatic iron and rule out other liver diseases. The Batts-Ludwig scoring system estimates ‘the proportion of hepatocytes that stain for iron’, where grades span from 1 to 4; the latter number representing pan-lobular iron deposition [49, 51]. Also, the historical hepatic iron index could be used for more accurate iron measurement [50].

Based on the iron distribution pattern, liver biopsy is useful for discerning conditions of secondary iron loading, as observed in about 50% cases of ARLD, NAFLD and viral hepatitis. For example, in these conditions, iron distribution is usually mild and occurs in hepatocytes and Kupffer cells, whereas in ferroportin disease (caused by mutation in the ferroportin gene that encodes for the cellular iron exporter), iron deposition is mostly in the reticuloendothelial system (includes macrophages and specialised endothelial cells) [49].

### Hepatocellular carcinoma (HCC)

With its increasing incidence in the last few years, HCC accounts for about 90% of primary liver cancers. It often develops following advanced cirrhosis. Liver biopsy becomes essential if HCC occurs in a non-cirrhotic patient, and biopsy can prevent the risk of misdiagnosis if imaging results have been inconclusive. Furthermore, it can differentiate HCC from other primary and secondary liver malignancies and enhance diagnosis beyond morphological evaluation by checking specific biomarkers of hepatocellular differentiation such as Arginase or bile salt export pump (BSEP). Indeed, tissue markers like glypican-3, glutamine synthetase and heat shock protein-70 can help characterise the hepatocellular lesion and differentiate between high grade dysplastic nodule and early HCC, when used in combination. In the future, liver biopsy could facilitate genetic and molecular analysis that enhance personalised and precision medicine, particularly for HCC in the context of prognostic stratification and identifying therapeutic targets [54].

Notably, AASLD does not recommend liver biopsy if the lesion is bigger than 1 cm and if two imaging studies reveal similar findings that confirm the cancer [54]. Availability of screening measures for HCC in CLD patients, ultrasonography, and reliance on HCC serum biomarkers like alpha-fetoprotein collectively reduce the need for liver biopsy. As such, using biopsy for HCC diagnosis and assessment remains controversial. One of the main reasons for this is needle tract tumour seeding, which can occur following HCC biopsy; its incidence being 2.7% [55].

## Repeat biopsy

Liver biopsy is one of the most reliable means of assessing a liver condition. Repeat liver biopsy is indeed capable of evaluating how much the disease has progressed or regressed over time. It is effective in determining the existence of comorbid liver conditions, in examining the efficacy of a particular treatment and in clarifying inconclusive findings from the 1st biopsy. While a decision needs to be made on whether to conduct the 1st liver biopsy, a decision also needs to be made after a successful disease diagnosis on whether to assess the effect of treatment via a follow-up biopsy post treatment or instead utilise non-invasive modalities such as ultrasound, MRI, or computerised tomography (CT) scan. These imaging approaches are usually conclusive.

After a disease has been established, the indications of repeat biopsy include failure of non-invasive modalities to show any alteration following treatment, (treatment failure) or disease remission, before the therapy is discontinued [56]. In a study of autoimmune hepatitis, clinical indications for repeat biopsy included diagnostic uncertainty, clinical deterioration, staging of disease, medication withdrawal or remission, and insufficient sample for analysis. Here, repeat biopsies led to modification of treatment in many patients [57]. Another study of autoimmune hepatitis revealed that the most common indications of repeat biopsy were increased liver function tests (increased transaminases) and indications related to treatment, while others underwent repeat biopsy for diagnostic clarification or to restage the hepatic fibrosis. In autoimmune hepatitis, the levels of aminotransferases do not always and reliably indicate inflammation and fibrosis progression. Hence, repeat biopsy is very important for proper evaluation of this liver condition. In this study, repeat biopsies helped assess disease progression in patients; these biopsies revealed improvement, worsening or no alteration in inflammation and helped establish whether there was fibrosis regression, progression, or stability. Disease management plans were altered following repeat biopsies [58].

Repeat liver biopsies have aided in the evaluation of other liver conditions too. For example, those on adults infected with hepatitis C virus revealed fibrosis progression with time, and those on untreated infected children showed increase in fibrosis or no histological progression of liver disease [59]. When the original biopsy failed to provide a reliable diagnosis, repeat biopsy was performed on cirrhotic patients and these were resultantly diagnosed with HCC [60]. Also, repeat liver biopsy helped assess the rate of fibrosis progression in untreated NASH patients [61]. Notably, even when the 1^st^ biopsy result can show normal or near normal result, there is a possibility of a liver condition being diagnosed in the follow-up biopsy or through other clinical data [62]. Collectively, these examples demonstrate the significance of repeat biopsy in evaluating liver diseases, and in making decisions related to treatment and disease management. Interestingly, the gap between 1st and repeat biopsy has been variable in different studies; 2 years [57], 6.1 ± 4.6 years [58] or about 4.3 years (range of 3.0–14.3 years) [61].

## Limitations and caveats of utilising liver biopsy for assessing CLDs

There is post-procedural pain, but this is usually not severe [8]. There are several other issues associated with liver biopsy. These include high cost, patient resistance, CLD aetiology, and the dependence of the accuracy of fibrosis staging on the length/quality of biopsy sample [9]. Other core limitations are discussed here.

### Invasive nature and risk of bleeding

Liver biopsies are invasive in nature. There can be severe bleeding following percutaneous liver biopsy and this can lead to haemodynamic instability [8]. The procedure may pose a risk of life-threatening complications such as haemorrhage, hypotension, infection, and injury to adjacent viscera and pneumothorax [63]. Although low, there is a risk of death with mortality rate of around 0.01% [9]. Apparently, while targeted biopsy has been linked with a higher risk of major complications [8], a study showed that the risks were similar [64]. Thus, AASLD recommends that liver biopsies should be carried out only if a definitive diagnosis (which cannot be ascertained in any other way apart from a biopsy) is likely to alter disease management or if there are abnormal liver tests of unknown aetiology, or if there is multiple parenchymal disease [11]. Liver biopsies are absolutely contraindicated if the patient presents increased risk of bleeding i.e. in cases with international normalised ratio (INR) > 1.5 or platelet count < 60,000/mm^3^ or if the patient has vascular tumours of the liver. Relative contraindications include the presence of ascites and morbid obesity. Both these make the procedure difficult and increase the risk of complications [12].

### Sampling error

Liver biopsy procedure extracts an extremely small portion from the liver and this portion is assumed to represent the entire liver. Notably, in several liver diseases such as NASH, the liver is not affected uniformly [65]. Therefore, a small sample obtained from a specific liver location cannot represent the pathological status of the entire liver. For example, laparoscopy-guided biopsies were conducted on patients affected with hepatitis C and samples were obtained from both right and left liver lobes of each patient. Two hepato-pathologists staged and graded the blinded samples using the Batts-Ludwig score. Results showed that 24.2% patients differed in at least one grade and 33.1% patients differed in at least one stage between the two liver lobes. Also, 14.5% patients were diagnosed with stage 3 fibrosis on one lobe, but with cirrhosis (stage 4) on the other lobe. Thus, these patients could have been underdiagnosed and they would not have known that they had cirrhosis [66]. Moreover, staging and grading can vary between samples even if these are obtained from the same lobe and are examined by the same assessor. For instance, in a study, individuals with NAFLD underwent biopsies where two separate histological samples were obtained from the same patient and from the same liver lobe. However, evaluation showed significant sampling error where 41% of the samples from the same lobe of the liver were staged differently by the same assessor [65]. These studies demonstrate the impact of sampling error on CLD assessment when using liver biopsies.

### Intra-observer and inter-observer variability

Interpretations of liver biopsies are subjective because they require pathologists to examine the qualitative features of the histological samples. This fosters intra-observer and inter-observer variability. However, a study that used Batts-Ludwig score reported near-perfect intra-observer agreement for staging and grading of liver biopsy samples (*k* ≥ 0.89 for each pathologist) [66]. Similarly, a study assessed inter-observer and intra-observer variability between four pathologists that used the METAVIR scoring system to evaluate liver samples from patients suffering from chronic viral hepatitis. Here, intra-observer agreement between pathologists was substantial (mean *k* ± SD: 0.77 ± 0.18). It was better than the inter-observer agreement, which was considered as moderate (mean *k* ± SD: 0.58 ± 0.26). The difference was attributed to the differences between the years of experience of the pathologists [67]. In another study, slight-to-moderate inter-observer agreements were observed when five pathologists used AHHS to assess histological samples from patients with ARLD. Here, the *k*-coefficient ranged from 0.20 to 0.52 for the individual histological features of alcoholic hepatitis [68]. These studies show that there can be substantial-to-near perfect intra-observer agreement and slight-to-moderate inter-observer agreement, thereby potentiating variability in disease staging and grading when examining the liver biopsy specimen.

## Summary

CLDs are associated with morbidity and mortality. Early diagnosis and treatment followed by appropriate disease monitoring and management can improve disease outcome. Although used less frequently than before, liver biopsy can play a pivotal role in diagnostic, therapeutic and prognostic processes and in monitoring the pathological stages of CLD.

To assess the liver biopsy specimen based on histology, various scoring systems have been proposed. The most implemented ones include the Ishak, METAVIR and Batts-Ludwig systems that determine the stage (level of fibrosis) and grade (level of necroinflammation), irrespective of disease aetiology.

Liver biopsy is considered as a gold standard for assessing viral hepatitis and NASH diagnosis. NAFLD requires biopsy for definitive diagnosis and to distinguish between simple steatosis and NASH. MAFLD is a newly coined term that is clinically more inclusive than NAFLD. It can be staged and graded like other CLDs. Liver biopsy is rarely needed for ARLD diagnosis and is not required anymore to diagnose haemochromatosis, although it is useful for determining cirrhosis. Liver biopsy can help distinguish HCC from other malignancies, but its usage for HCC evaluation is controversial.

The invasive nature of liver biopsy can cause complications such as haemorrhage, hypotension, pneumothorax and even death. Sampling error is one of the limitations of this approach because biopsies allow the examination of a very small region of the liver, thereby giving misleading results. This can be further confounded by intra-observer and inter-observer variabilities.
